# Hypocoagulable Tendency on Thromboelastometry Associated With Severity and Anticoagulation Timing in Pediatric Septic Shock: A Prospective Observational Study

**DOI:** 10.3389/fped.2021.676565

**Published:** 2021-06-02

**Authors:** Ta Anh Tuan, Nguyen Thi Thu Ha, Tran Dang Xoay, Tran Thi Kieu My, Luong Thi Nghiem, Tran Minh Dien

**Affiliations:** ^1^Pediatric Intensive Care Unit, Vietnam National Children's Hospital, Hanoi, Vietnam; ^2^University of Medicine and Pharmacy, Vietnam National University, Hanoi, Vietnam; ^3^Division of Hematology, Hanoi Medical University, Hanoi, Vietnam; ^4^Hematology Department, Vietnam National Children's Hospital, Hanoi, Vietnam; ^5^Surgical Intensive Care Unit, Vietnam National Children's Hospital, Hanoi, Vietnam

**Keywords:** hypocoagulable, pediatric, septic shock, thromboelastometry, PELOD-2, PRISM-III

## Abstract

**Objective:** To identify whether coagulation profiles using thromboelastometry are associated with outcomes in pediatric septic shock. The primary outcomes were the development of disseminated intravascular coagulation (DIC) and the severity of the pediatric intensive care unit (PICU) existing scoring systems, while the secondary outcome was hospital mortality. This study aimed to contribute to current findings of the limitations of conventional tests in determining the optimal timing of anticoagulation in sepsis.

**Design:** A prospective, observational study conducted between August 2019 and August 2020.

**Setting:** PICU at a pediatric tertiary hospital in Hanoi, Vietnam.

**Patients:** Fifty-five pediatric patients who met the septic shock criteria were enrolled.

**Measurements and Main Results:** Fifty-five patients with septic shock were recruited. At the time of diagnosis, thromboelastometry revealed normocoagulability, hypercoagulability, and hypocoagulability in 29, 29, and 42% of the patients, respectively (*p* > 0.05); however, most patients in the overt DIC and non-survival groups progressed to hypocoagulability (82 and 64%, respectively). The overt DIC, PELOD-2 > 8, PRISM-III > 11, and non-survival group had a significant hypocoagulable tendency according to thromboelastometry parameters [prolonged clotting time (CT) and clot formation time (CFT); and reduced α-angle (α), maximum clot firmness (MCF), thrombodynamic potential index (TPI)] compared to the non-overt DIC, PELOD-2 ≤ 8, PRISM-III score ≤ 11 and survival group (*p* < 0.05). Conventional parameters between the normocoagulable and hypercoagulable groups were not different (*p* > 0.05). Hypocoagulability was characterized by lower platelet count and fibrinogen level, higher prolonged prothrombin time (PT), international normalized ratio (INR), and activated partial thromboplastin time (APTT), and higher D-dimer level than in hypercoagulability (*p* < 0.05). Hypocoagulable tendency on thromboelastometry had a higher hazard at a PT > 16.1 s [area under the curve (AUC) = 0.747, odds ratio (OR) = 10.5, *p* = 0.002], INR > 1.4 (AUC = 0.754, OR = 6.9, *p* = 0.001), fibrinogen <3.3 g/L (AUC = 0.728, OR = 9.9, *p* = 0.004), and D-dimer > 3,863 ng/mL (AUC = 0.728, OR = 6.7, *p* = 0.004).

**Conclusions:** Hypocoagulable tendency using thromboelastometry is associated with the severity of septic shock. Conventional coagulation tests may fail to detect hypercoagulability, which is crucial in determining anticoagulation timing.

## Introduction

Sepsis-induced coagulopathy (SIC) is initially triggered by the combination of procoagulant upregulation, endogenous anticoagulant downregulation, and fibrinolytic impairment. These mechanisms are beneficial host responses that contribute to a hypercoagulable state, which is termed “immunothrombosis”—a defense against the spread of pathogens into the nearby tissues or systemic circulation at an early stage of sepsis (1–3). However, its dysregulation leads to overwhelming activation of coagulation that induces microthrombosis, particularly disseminated intravascular coagulation (DIC) and multiple organ dysfunction syndrome (MODS). The ongoing excessive consumption of coagulation factors ultimately results in a hypocoagulable state with a high risk of severe hemorrhagic complications (2, 4, 5). Previous studies have reported that the early phase of sepsis is characterized by hypercoagulability, whereas the later stages (severe sepsis or septic shock) trend toward hypocoagulability and are associated with increased morbidity and mortality (1, 6–12). Therefore, proper identification of the coagulation profile is pivotal for appropriate treatment and prognosis. The timing of coagulation-targeting therapy at the hypercoagulable stage may reduce the risk of disseminative thrombosis and progression of MODS. In contrast, blood transfusion therapy is indicated during hypocoagulability with a high risk of bleeding because of the depleted components of the coagulation system (2, 13). However, the current standards for hemostatic assessment are generally based on conventional parameters with several limitations. As they are performed using plasma, the function of cell components (such as the contribution of platelets to thrombosis) in the coagulation system is bypassed. Additionally, these parameters are not reflective of the *in vivo* hemostatic process and do not provide qualitative or functional data (1, 14). Moreover, their inability to detect hypercoagulable status and fibrinolytic activity has been revealed (15, 16). Hence, methodological improvements in thromboelastography (TEG) or rotational thromboelastometry (ROTEM) have been widely utilized as point-of-care tests for optimal hemostatic resuscitation in patients with sepsis. These tests measure dynamic global clotting and overcome limitations of the conventional assay in discriminating between hyper- and hypocoagulability (10–12, 17). Hypocoagulable profile in viscoelastic parameters is associated with poor outcomes (11); therefore, clinicians should monitor the patients more strictly and adopt adequate diagnostic and therapeutic strategies earlier. Furthermore, the efficacy of the viscoelastic test for the guidance of blood transfusion practice has been reported (15, 16). Prior studies using global clotting assay proved its ability to evaluate coagulation and prognosis. However, these data were mainly obtained in adults and cases of non-septic shock, and the broader use of ROTEM remained controversial (8, 15); therefore, additional studies are required. Our study aimed to evaluate hemostatic disturbance using the ROTEM assay and identify the association between these parameters and the severity of septic shock in pediatric patients. Additionally, we aimed to contribute to the current findings on the limitations of conventional coagulation tests in optimal anticoagulation timing in sepsis.

## Materials and Methods

### Ethics Approval

Ethics approval for our study was obtained from the Institutional Review Board of Vietnam National Children's Hospital (approval no. 197-BVNTW-VNCSKTE).

### Patients

This was a single-center prospective observational study from August 2019 to August 2020 at the pediatric intensive care unit (PICU) of a tertiary hospital in Vietnam. Patients aged 1 month−18 years with septic shock, based on the criteria of the International Sepsis Definitions Conference 2005, were recruited (18). Patients with a history of coagulopathy, those undergoing anticoagulant treatment, or those who died within 24 h of admission were excluded from this study.

Patient demographic, clinical, and laboratory variables were collected. Demographic variables included age, sex, weight, and underlying conditions. Clinical variables included temperature, heart rate, ventilatory condition, mean arterial pressure, consciousness level evaluated according to the Glasgow Coma Scale (19), and hemorrhagic or thrombotic events. Laboratory variables included white blood count (×10^9^/L), hemoglobin (g/L), C-reactive protein (mg/L), lactate level (mmol/L), urea (mmol/L), creatinine (μmol/L), aspartate aminotransferase (IU/L), alanine aminotransferase (IU/L), albumin (g/L), protein (g/L), direct and indirect bilirubin (mg/dL), and blood culture results. Pediatric Risk of Mortality III (PRISM-III) and Pediatric Logistic Organ Dysfunction-2 (PELOD-2) scores at 24-hour after septic shock diagnosis were calculated (20, 21).

### Conventional Coagulation Parameters and DIC Score

The conventional coagulation parameters at the time of diagnosis of septic shock were platelet count (PLT) (×10^9^/L) [using K-4500 hematology analyzer (Sysmex, Kobe, Japan)], prothrombin time (PT) (s), international normalized ratio (INR), activated partial thromboplastin time (APTT) (s), fibrinogen (g/L), and D-dimer (ng/mL FEU) [using ACL TOP 750-CTS coagulation analyzer (Instrument Laboratory, American)]. DIC score was calculated according to the International Society of Thrombosis and Hemostasis (ISTH) criteria, 2001: (1) PLT count from 50 to 100 ×10^9^/L (1 point), <50 ×10^9^/L (2 points); (2) INR from 1.4 to 2.3 (1 point), >2.3 (2 points); (3) fibrinogen <1 g/L (1 point); and (4) D-dimer from 399 to 4,000 ng/mL (2 points), >4,000 ng/mL (3 points). Patients had overt-DIC if the DIC score was ≥5 (4).

### Rotation Thromboelastometry

**Figure 1:**
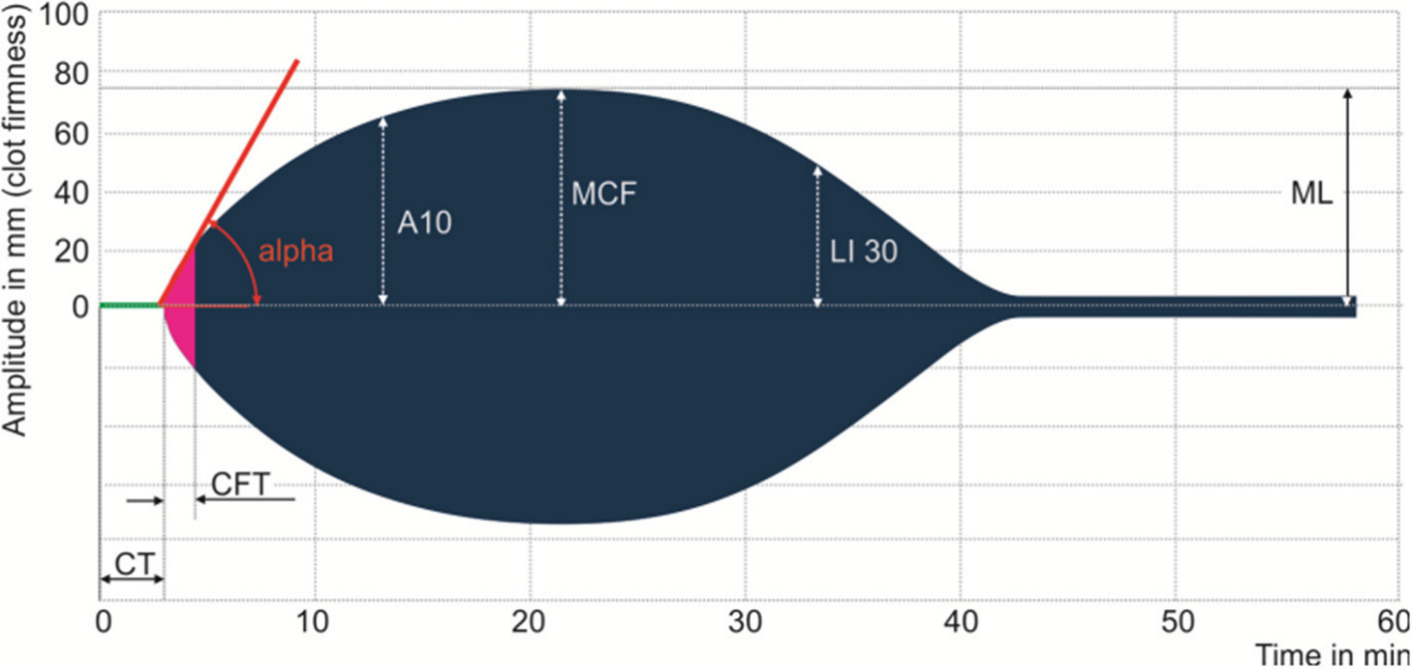
Main parameters of the rotational thromboelastometry test. Clotting time (CT), clot formation time (CFT), alpha angle (ALP), the amplitude after 10 min after CT, maximum clot firmness (MCF), clot lysis index at 30 min after CT (LI30), and maximum lysis (ML).

Samples of whole blood anticoagulated with 3.8% sodium citrate were collected at the time of diagnosis and analyzed with ROTEM delta analyzer (ROTEM®, TEM International GmbH, Munich, Germany). Four main tests were performed: EXTEM (the extrinsic pathway); INTEM (the intrinsic pathway); FIBTEM (the contribution of fibrinogen to the clot); and APTEM (assessment of hyperfibrinolysis) (22, 23). Each test records the kinetic changes in a sample of whole blood during clot formation and when the clot retracts and/or lyses. As external pathway activation plays a crucial role in SIC, our study focused on EXTEM parameters, including clotting time (CT)—time to thrombin generation, clot formation time (CFT), and α-angle (α)—fibrin polymerization and clot formation, maximum clot firmness (MCF)—the measure of maximum clot amplitude describing clot stability and thrombodynamic potential index (TPI) calculated by 30^*^(100^*^MCF)/CFT^*^(100-MCF) describing global coagulation. Three coagulation profiles were categorized on EXTEM, including normocoagulability, hypercoagulability, and hypocoagulability. Hypercoagulability was defined as at least one of shortened CT or CFT, elevated α, MCF, or TPI. Hypocoagulability was defined as at least one of prolonged CT or CFT, reduced α, MCF, or TPI. The reference range of the aforementioned EXTEM parameters were determined by a multicenter investigation of healthy volunteers (24, 25). Figure 1 is a diagram illustrating the main parameters of the rotational thromboelastomatry test.

### Statistical Analysis

Statistical analyses were performed using SPSS software version 20.0 software (IBM Corporation, Armonk, NY). Categorical variables are described as frequency and percentage, whereas continuous variables are expressed as medians and interquartile ranges (IQRs). We used the Mann-Whitney U test to compare the mean of non-parametric variables between the two groups; Chi-square test was used to test the equality of independent proportions. The receiver operating characteristic (ROC) curve with the Younden index was used to identify the performance and cutoff point of conventional parameters on discrimination in coagulation profiles. Area under the ROC (AUC) curve values that fell between 0.7 and 0.8 were acceptable, between 0.8 and 0.9 were excellent, and >0.9 were outstanding (26). A two-sided *p*-value of < 0.05 was considered statistically significant.

## Results

### Patients

Fifty-five patients (31 boys and 24 girls) were recruited in this study. The median age was 7 (range: 1–205) months, and 13% had comorbidities. The frequency of clinical bleeding and thrombosis was 20 and 2%, respectively. Of the total, 31% presented with positive blood cultures, and the most prevalent organisms isolated were *Streptococcus pneumonia, Staphylococcus aureus*, and *Escherichia coli*. The median PELOD-2 score and PRISM-III scores were 8 and 11, respectively. At the time of diagnosis, the prevalence of overt-DIC was 20%. The mortality rate of the overt-DIC group was 55%, while the overall mortality rate was 31% (Table 1).

**Table 1:** Patient characteristics.

**Characteristics**	**Value (*n* = 55)**
**Demographic data**	
Age, months (Median, range)	7 (1–205)
Male sex (*n*, %)	31 (56%)
Underlying conditions (*n*, %)	7 (13%)
**Clinical presentations of hemostatic abnormalities (*****n*****, %)**
Bleeding	11 (20%)
Thrombosis	1 (2%)
Positive blood culture (*n*, %)	17 (31%)
*Streptococcus pneumoniae*	4 (24%)
*Staphylococcus aureus*	4 (24%)
*Escherichia coli*	4 (24%)
*Enterobacter cloacae*	1 (6%)
*Burkholderia pseudomallei*	1 (6%)
*Burkholderia cepacia*	1 (6%)
*Pseudomonas aeruginosa*	1 (6%)
*Klebsiella pneumoniae*	1 (6%)
*Kodamaea ohmeri*	1 (6%)
VIS score (*n*, %)	55 (100%)
At 24-h post PICU admission (Median, IQR)	20 (7-43)
PELOD-2 score (Median, IQR)	8 (6-11)
PRISM-III score (Median, IQR)	11 (7-18)
Overt DIC at diagnostic time (*n*, %)	11 (20%)
Non-invasive mechanical ventilation (*n*, %)	7 (13%)
Invasive mechanical ventilation (*n*, %)	48 (87%)
Length of invasive mechanical ventilation (days), median (IQR)	5 (3-7)
PICU length of stay (days), median (IQR)	14 (5-20)
Mortality (*n*, %)	17 (31%)

*Data are presented as median (IQR: Q1–Q3) or number (%)*.

*IQR, Interquartile Range; PELOD-2, Pediatric Logistic Organ Dysfunction-2; PRISM-III, Pediatric Risk of Mortality Score III; DIC, Disseminated intravascular coagulation; PICU, pediatric intensive care unit*.

### Conventional Coagulation Data

Supplementary Table enlists that the overt-DIC, PELOD-2 > 8, and PRISM-III > 11 group had lower platelet count and fibrinogen level, more prolonged PT, INR, APTT, and higher D-dimer than their counterparts, the non-overt DIC, PELOD-2 ≤ 8, and PRISM-III ≤ 11 group (*p* < 0.05). The difference in these parameters between the survivors and non-survivors was not significant (*p* > 0.05).

### Rotational Thromboelastometry Data

**Figure 2:**
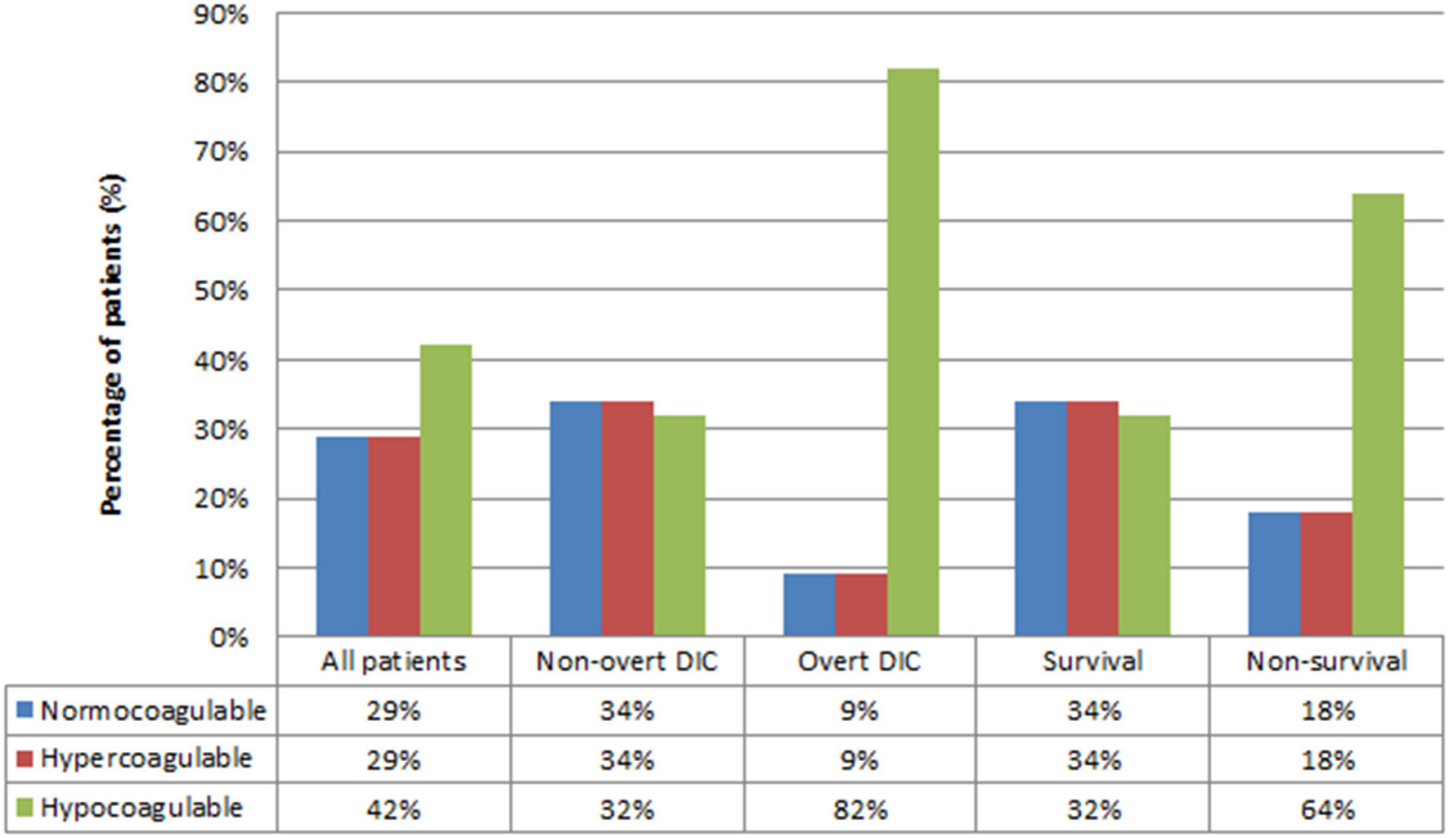
Coagulation profiles based on rotational thromboelastometry test. DIC, Disseminated intravascular coagulation. *P*-value for Chi-square test of three coagulation profiles for all patients = 0.41, non-overt DIC = 0.978, overt-DIC = 0.003, survival = 0.974, non-survival = 0.023.

Coagulation profiles on EXTEM are displayed in Figure 2. Overall, the proportion of coagulation profiles was not different (*p* > 0.05), with 29, 29, and 42% being normocoagulable, hypercoagulable, and hypocoagulable, respectively. However, subgroup analyses revealed that most patients with overt-DIC and non-survival progressed to the hypocoagulable state at the time of diagnosis [*p* < 0.05 (82 and 64%, respectively)].

The overt-DIC, PELOD-2 > 8, PRISM-III > 11, and non-survival group had significant hypocoagulable tendencies in the studied parameters (prolonged CT, CFT and reduced α, MCF, TPI) compared with those of the non-overt DIC, PELOD-2 ≤ 8, PRISM-III ≤ 11, and survival group (*p* < 0.05) (Supplementary Table).

### Conventional Coagulation Tests Failed to Detect the Hypercoagulable

In this study, we compared the conventional parameters among subgroups of coagulation profiles on EXTEM (normocoagulability, hypercoagulability, and hypocoagulability) (Table 2). An insignificant difference in most parameters (PT, INR, APTT, fibrinogen, D-Dimer) was observed between the normocoagulable and hypercoagulable groups (*p* > 0.05). Hypocoagulability was associated with a lower platelet count and fibrinogen level, but more prolonged PT, INR, APTT, and higher D-dimer level than hypercoagulability (*p* < 0.05).

**Table 2:** Performance of conventional parameters in the discrimination of coagulation status based on thromboelastometry.

**Parameters(Median, IQR)**	**Normocoagulable (*n* = 16)**	**Hypercoagulable(*n* = 16)**	**Hypocoagulable (*n* = 23)**	***p*^a^**	***p*^b^**	***p*^c^**
PLT (×10^9^/L)	227 (109–365)	474 (300–549)	177 (66–253)	**0.009**	**<0.001**	0.242
PT (s)	15 (14–29.6)	13.9 (12.7–18.7)	20 (16.2–26.3)	0.356	**0.003**	**0.028**
INR	1.3 (1.1–1.6)	1.2 (1–1.5)	1.6 (1.4–2.4)	0.327	**0.003**	**0.019**
APTT (s)	40.7 (37–45.3)	36.9 (31.8–51.3)	48.3 (38–65.6)	0.546	**0.050**	0.056
Fibrinogen (g/L)	4 (3.7–5)	3.9 (2.9–5.4)	2.6 (1.5–4.2)	0.821	**0.035**	**0.007**
D-Dimer (ng/mL)	2,664 (1,954–5,281)	2,096 (974–3,381)	6,935 (2,343–17,172)	0.200	**0.006**	**0.044**

*Data are presented as median (IQR: Q1–Q3)*.

a, b, c *Mann–Whitney U-test comparing non-parametric variables between two subgroups: normocoagulable vs. hypercoagulable; hypercoagulable vs. hypocoagulable; normocoagulable vs. hypocoagulable, respectively. Bold values indicate the statistically significant (p-value < 0.05)*.

*IQR, Interquartile range; PLT, Platelet; PT, Prothrombin Time; INR, International Normalized Ratio; APTT, Activated Partial Thromboplastin Time*.

Further analyses revealed that the hypocoagulable tendency on thromboelastometry had a higher hazard at a PT > 16.1 s [AUC = 0.747, odds ratio (OR) = 10.5, *p* = 0.002], INR > 1.4 (AUC = 0.754, OR = 6.9, *p* = 0.001), fibrinogen <3.3 g/L (AUC = 0.728, OR = 9.9, *p* = 0.004), and D-dimer > 3,863 ng/mL (AUC = 0.728, OR = 6.7, *p* = 0.004) (Table 3).

**Table 3:** Cutoff point of conventional parameters in the discrimination of hypocoagulable phase (based on rotational thromboelastometry).

**Parameters**	**Normo/hypercoagulable**	**Hypocoagulable**	**AUC**	**Cutoff**	***p*-value**	**OR**
**(Mean, IQR)**	**(*n* = 32)**	**(*n* = 23)**	**(95%CI)**	**(Se–Sp)**		**(95% CI)**
PT (s)	14.7 (12.9–19.6)	20 (16.2–26.3)	0.747 (0.615–0.878)	16.1 (82.6–68.7)	**0.002**	10.5 (2.8–38.8)
INR	1.2 (1–1.5)	1.6 (1.4–2.4)	0.754 (0.624–0.884)	1.4 (78.3–68.7)	**0.001**	6.9 (2–23.5)
Fibrinogen (g/L)	3.9 (3.6–5.3)	2.6 (1.5–4.2)	0.728 (0.582–0.874)	3.3 (69.6–81.2)	**0.004**	9.9 (2.8–34.8)
D-dimer (ng/mL)	2,371 (1,617–3,801)	6,935 (2,343–17,172)	0.728 (0.588–0.867)	3,863 (65.2– 78.1)	**0.004**	6.7 (2–22.2)

*AUC, Area Under the Receiver Operating Characteristic Curve; CI, Confidence Interval; Se, Sensitivity; Sp, Specificity; OR, odds ratio. Bold values indicate the statistically significant (p-value < 0.05)*.

*IQR, Interquartile range; PLT, Platelet; PT, Prothrombin Time; INR, International Normalized Ratio; APTT, Activated Partial Thromboplastin Time*.

## Discussion

This study determined the relationship between hemostatic disturbance from viscoelastic assay and severity in 55 pediatric patients with septic shock at the PICU of the Vietnam National Children's Hospital. To the best of our knowledge, this is the first report from a developing country describing the association between hypocoagulation profile based on thromboelastometry and the severity of septic shock in a pediatric population; additionally, this study further strengthened the findings of previous reports on the drawbacks of conventional coagulation tests.

Previous studies in adults reported the detection of hypercoagulability on viscoelastic assay as ranging from 30 to 100% (17). Hypercoagulability was observed in the early stage of sepsis, consistent with the aforementioned hypothesis of “immunothrombosis” defense (1, 3, 15). The development of hypocoagulability is commonly associated with the increasing severity of each stage (severe sepsis and septic shock) (12, 15, 16, 27). Previous studies observed hemostatic response by a rapid increase in clot mass development (with higher α and MCF), possibly indicating a hypercoagulable phase and potential thromboembolic risk in the early stage of sepsis followed by the progression of hypocoagulable trends (with prolonged CT and CFT) in the later phase (6, 28). In a previous study on adult sepsis, TEG profile of the septic shock group at the time of intensive care unit (ICU) entry and 6 h after ICU entry significantly tended to be more hypocoagulable than that of the sepsis group (12). Similarly, our result showed that most patients were in the hypocoagulable stage on EXTEM at the time of septic shock diagnosis; however, the difference among profiles was insignificant (*p* > 0.05) (Figure 2).

Interestingly, the highest prevalence of hypocoagulability was observed in the overt-DIC and non-survival groups on subgroup analyses in this study (Figure 2). The non-overt DIC group was mostly hypercoagulable, while progression to hypocoagulability was observed in the overt-DIC group (22). We hypothesized that most cases who met the overt-DIC criteria developed hypocoagulability secondary to the depletion of coagulation factors; therefore, using the ISTH scoring system may delay the early-stage diagnosis of septic coagulopathy. Other studies, mainly in adult populations, also revealed that hypocoagulability was characterized by high CT and CFT and low MCF in patients with overt-DIC compared to patients with non-overt DIC (22, 29, 30). Depleted coagulation factors during DIC can be a reasonable explanation for the prolonged CT and CFT. Lower MCF in overt DIC was induced by thrombocytopenia and hypofibrinogenemia, which are two main factors that influence the firmness of clots. Similarly, various studies reported that hypocoagulability was associated with higher consumption of blood products and lower survival. Previous studies identifying parameters with the highest prognostic value of mortality have been reported; however, the results remain unclear (9–12, 17, 31, 32). In addition, the data in the pediatric population were limited; hence, further investigations are warranted to clarify this issue. Further, our analyses revealed that increasing severities (over-DIC, high PELOD-2 score, high PRISM-III score, non-survival) were associated with more hypocoagulable tendency in each EXTEM parameter (prolonged CT, CFT, but lower α, MCF, TPI) compared with the less severe groups (non-overt DIC, low PELOD-2 score, low PRISM-III score, survival) (Supplementary Table). To the best of our knowledge, this is the first study comparing ROTEM parameters between the severity subgroups of the existing scoring system in pediatric patients. In previous septic studies in adults based on TEG/ROTEM, a higher sequential organ failure assessment (SOFA) score indicated a more hypocoagulable state than a lower SOFA score (9, 16, 33).

Theoretically, sepsis-associated coagulopathy may lead to a variable clinical picture, ranging from hypercoagulopathy with microthrombosis to an increase in bleeding complications because of the secondary consumption of coagulation factors and platelets (1, 3, 4). Therefore, identifying the coagulation profile is pivotal in timing anticoagulant therapy to both preserve the defense function of the hemostatic system and to avoid the harmful effects of overwhelming activation of coagulation. In fact, conventional coagulation tests are simply to be routinely utilized but are incapable of distinguishing the stages of hypercoagulability (15, 16). Table 2 shows no difference in most standard parameters (PT, INR, APTT, fibrinogen, D-dimer) between the normocoagulable and the hypercoagulable states. Although prolonged PT, INR, and APTT may occur, the profile on EXTEM would remain normocoagulable (Table 2). Prolonged PT, INR, and APTT, but high D-dimer, can lead to the misinterpretation of hyper- or hypocoagulability. In 2019, Saini et al. reported that hypercoagulability on TEG was more common than hypocoagulability; in contrast, hypocoagulability was more frequently observed on standard assays in children with severe sepsis (15). This can be explained as they rely on serum portions of the clotting cascade in isolation, and the PT and APTT only reflect clot formation partly. Therefore, a decrease in PT (%) and prolongation of APTT are poor predictors of hemorrhage, which may overestimate the need for transfusion therapy (34, 35). Moreover, although patients had normal platelet count and fibrinogen level, the EXTEM may display a hypocoagulable profile (Table 2). This may be explained by the impaired platelet aggregation and fibrinogen function, which frequently manifest in sepsis (11, 12, 36, 37). Thus far, the contribution of both quantitative and functional platelets to clot strength was involved in ROTEM but eliminated in the standard assay. Interestingly, Table 3 shows that fibrinogen <3.3 g/L (AUC = 0.728, OR = 9.9, *p* = 0.004) and D-dimer > 3,863 ng/mL (AUC = 0.728, OR = 6.7, *p* = 0.004) were the cutoff points corresponding with greater hazard of hypocoagulability on EXTEM. However, fibrinogen <1 (g/L) and D-dimer > 4,000 (ng/mL) are the thresholds according to the ISTH criteria for 2 and 3 points, respectively (4). We strengthened the aforementioned hypothesis that using the ITSH criteria may delay the diagnosis of early phase or a hypercoagulable stage for the indication of anticoagulant therapy. Several studies in adult patients reported that the existing DIC scoring systems (such as ISTH, JAAM - Japanese Association for Acute Medicine) are valuable for fatal prognosis but late for timing diagnosis of “early asymptomatic DIC,” “pre-DIC,” “non-overt DIC,” and “compensated DIC” (2, 38–40). Currently, the concept of SIC score combining platelet count, PT ratio, and total SOFA was recognized as the new tool for early coagulopathy detection, although with low specificity (39–41). This implies that DIC is initially characterized by widespread microvascular thrombosis induced in impaired organs; therefore, SOFA score describing both the number and the severity of organ dysfunction may allow early identification of the hypercoagulable stage for timely anticoagulant therapy. Nevertheless, further studies are required to investigate this issue on pediatric sepsis.

Our study has some limitations. A small number of participants were included from a single-center, and there was no existing standard definition of hypo- and hypercoagulability for ROTEM (17). Moreover, this study collected the coagulation parameters at a single time and did not evaluate their variations during the subsequent days. However, this may be beneficial as we can eliminate the effects of blood transfusion or anticoagulant therapy during treatment on hemostatic parameters. Nonetheless, further well-designed investigations should be undertaken to improve upon the findings of our study.

## Conclusions

Hypocoagulable profile in patients with pediatric septic shock was associated with more severe conditions of the disease. Conventional tests may fail to detect hypercoagulability—the early stage of SIC, which is crucial in determining anticoagulation timing.

## Data Availability Statement

The original contributions presented in the study are included in the article/Supplementary Material, further inquiries can be directed to the corresponding author/s.

## Ethics Statement

The studies involving human participants were reviewed and approved by Institutional Review Board of Vietnam National Children's Hospital (approval no. 197-BVNTW-VNCSKTE). Written informed consent to participate in this study was provided by the participants' legal guardian/next of kin.

## Author Contributions

TT, NH, TX, TM, and TD contributed to the conception and design of the study. TT, LN, and NH collected the clinical information. NH wrote the first draft of the manuscript. TT, NH, TX, and TM wrote sections of the manuscript. NH and LN enrolled the patients, performed the coagulation test, and reviewed the manuscript. All authors have revised, read, and approved the final version of the manuscript.

## Conflict of Interest

The authors declare that the research was conducted in the absence of any commercial or financial relationships that could be construed as a potential conflict of interest.
