# Supplementary material for: Hypocoagulable Tendency on Thromboelastometry Associated With Severity and Anticoagulation Timing in Pediatric Septic Shock: A Prospective Observational Study
Source: Front Pediatr. 2021 Jun 2;9:676565. doi: 10.3389/fped.2021.676565 (PMC8206499; doi:10.3389/fped.2021.676565)
Supplement: Supplementary file 1 [file Table_1.docx]

## Supplementary Table. Characteristics of coagulation parameters in pediatric septic shock

| **Parameters**  *(Median, IQR)* | **Total**  *(n = 56)* | **n–DIC**  *(n = 44)* | **oDIC**  *(n = 11)* | **PELOD-2 ≤8**  *(n=33)* | **PELOD-2 >8**  *(n=22)* | **PRISM-III ≤11**  *(n=29)* | **PRISM-III >11**  *(n=26)* | **Survival**  *(n = 38)* | **Non–survival**  *(n = 17)* | **p-value^d^** | **p-value^e^** | **p-value^f^** | **p-value^g^** |
| --- | --- | --- | --- | --- | --- | --- | --- | --- | --- | --- | --- | --- | --- |
| PLT *(x10^9^/L)* | 245  (112–452) | 282  (180–485) | 66  (51–112) | 327  (218–533) | 107  (56.5–242.8) | 357  (232.5–533) | 138.5  (64.3–257.8) | 260  (167–496) | 147  (63–326) | ***<0.001*** | ***<0.001*** | ***<0.001*** | 0.073 |
| PT *(s)* | 16.2  (13.7–21.4) | 15  (13.5–19.6) | 23.4  (20–28.6) | 14.7  (13.5–19.1) | 20.1  (15.9–28.2) | 14  (13.3–17.3) | 20.1  (16.7–28.2) | 15.3  (13.7–20.1) | 19.6  (13.7–28.3) | ***<0.001*** | ***0.004*** | ***<0.001*** | 0.122 |
| INR | 1.4  (1.2–1.8) | 1.3  (1.1–1.6) | 2.1  (1.5–2.6) | 1.3  (1.1–1.5) | 1.6  (1.4–2.6) | 1.2  (1–1.4) | 1.7  (1.5–2.6) | 1.3  (1.1–1.6) | 1.5  (1.3–2.6) | ***0.001*** | ***0.003*** | ***<0.001*** | 0.096 |
| APTT *(s)* | 41.2  (36.7–51.7) | 39.2  (35.1–46.6) | 55.7  (45.2–78.9) | 37.3  (32.4–44) | 49.8  (42.5–60.6) | 38.1  (34.2–45) | 47.5  (37.4–57.9) | 40.6  (36.2–47.8) | 48.3  (34.4–57.9) | ***0.007*** | ***<0.001*** | ***0.025*** | 0.362 |
| Fibrinogen *(g/L)* | 3.8  (2.2–5) | 3.9  (2.8–5.4) | 1.5  (0.9–3.1) | 3.9  (2.8–5.2) | 3.1  (1.6–4.7) | 4  (3.6–5.4) | 2.7  (1.6–4.3) | 3.9  (2.7–5) | 3.1  (1.5–4.8) | ***<0.001*** | 0.101 | ***0.007*** | 0.169 |
| D–Dimer *(ng/mL)* | 2905  (1917–7822) | 2594  (1617–5565.8) | 8829  (4175–84548) | 2145  (1501–5376.5) | 4990.5  (2845–16900) | 2275  (1434–3591) | 5514.5  (2294–17438) | 2841  (1924.5–6225.8) | 3549  (1867–10988) | ***0.002*** | ***0.006*** | ***0.001*** | 0.771 |
| CT *(s)* | 65  (54–80) | 61  (49–71.5) | 83  (69–422) | 57  (45–69.5) | 80  (65–102.3) | 58  (48–96.5) | 69.5  (60.3–93.8) | 61.5  (51–70.5) | 80  (63–121) | ***<0.001*** | ***<0.001*** | ***0.002*** | ***0.007*** |
| CFT *(s)* | 90  (60–173) | 76.5  (51.3–130) | 306  (173–453) | 69  (48.5–131.5) | 134  (84.5–450.8) | 61  (48–96.5) | 154  (106.5–422.3) | 70  (57.5–126.3) | 195  (98.5–451.5) | ***<0.001*** | ***0.001*** | ***<0.001*** | ***0.004*** |
| α *(^o^)* | 75  (62–80) | 76  (69–80) | 52  (30–73) | 77  (69–80) | 68  (39.5–75.5) | 78  (73.5–80.5) | 68  (47.8–74.3) | 76  (69–80) | 61  (38–76) | ***0.001*** | ***0.001*** | ***<0.001*** | ***0.009*** |
| MCF *(mm)* | 59  (47–68) | 64.5  (55–69) | 42  (28–50) | 66  (54.5–70.5) | 52.5  (32.3–61.8) | 67  (57.5–71) | 51.5  (36–58.5) | 65.5  (53.3–69.3) | 49  (34.5–60) | ***<0.001*** | ***0.002*** | ***<0.001*** | ***0.006*** |
| TPI | 46 (15–114) | 67.5  (28–121.8) | 9  (3–17) | 85  (25–149) | 28  (4–56.5) | 105  (51–163.5) | 21  (4–42.3) | 78.5  (28–124.5) | 15  (4–46.5) | ***<0.001*** | ***0.001*** | ***<0.001*** | ***0.008*** |

Data are presented by median (IQR: Q1–Q3).

d, e, f, g: Mann–Whitney U test comparing non–parametric variables between two subgroups: nDIC versus oDIC; PELOD–2 ≤8 score versus PELOD–2 >8 score; PRISM-III ≤11 score versus PRISM-III >11, survival versus non–survival, respectively. Our study used median values as cut–off point of PELOD-2 and PRISM-III score. A p–value less than 0.05 is statistically significant. IQR, Interquartile Range.

PLT, Platelet; PT, Prothrombin Time; INR, International Normalized Ratio; APTT, Activated Partial Thromboplastin Time; CT, Clotting Time; CFT, Clot Formation Time; α, Anpha-angle; MCF, Maximum Clot Firmness; TPI, Thrombodynamic Potential Index; oDIC, Overt Disseminated Intravascular Coagulation with DIC score ≥5, n–DIC, Non–overt Disseminated Intravascular Coagulation with DIC score <5 according to ISTH criteria in 2001 (4); PELOD-2, Pediatric Logistic Organ Dysfunction–2; PRISM-III, Pediatric Risk of Mortality Score III.
