# Supplementary material for: Hypocoagulable Tendency on Thromboelastometry Associated With Severity and Anticoagulation Timing in Pediatric Septic Shock: A Prospective Observational Study
Source: Front Pediatr. 2021 Jun 2;9:676565. doi: 10.3389/fped.2021.676565 (PMC8206499; doi:10.3389/fped.2021.676565)
Supplement: Supplementary file 2 [file Table_2.docx]

Supplementary Material

**Supplementary Table. Characteristics of coagulation parameters in pediatric septic shock**

d, e, f, g: Mann–Whitney U test comparing non–parametric variables between two subgroups: nDIC versus oDIC; PELOD–2 ≤8 score versus PELOD–2 >8 score; PRISM-III ≤11 score versus PRISM-III >11, survival versus non–survival, respectively. Our study used median values as cut–off point of PELOD-2 and PRISM-III score. A p–value less than 0.05 is statistically significant.

PLT, Platelet; PT, Prothrombin Time; INR, International Normalized Ratio; APTT, Activated Partial Thromboplastin Time; CT, Clotting Time; CFT, Clot Formation Time; α, Anpha-angle; MCF, Maximum Clot Firmness; TPI, Thrombodynamic Potential Index; oDIC, Overt Disseminated Intravascular Coagulation with DIC score ≥5, n–DIC, Non–overt Disseminated Intravascular Coagulation with DIC score <5 according to ISTH criteria in 2001 (4); PELOD-2, Pediatric Logistic Organ Dysfunction–2; PRISM-III, Pediatric Risk of Mortality Score III.
